# Development and reporting of a specialised antenatal music therapy intervention using the MRC framework and TIDieR checklist

**DOI:** 10.3389/fpsyt.2026.1758640

**Published:** 2026-02-09

**Authors:** Pui Sze Cheung, Sylvia Murphy Tighe, Mas Mahady Mohamad, Triona McCaffrey

**Affiliations:** 1 Health Research Institute, University of Limerick, Limerick, Ireland; 2 Specialist Perinatal Mental Health Service, University Maternity Hospital Limerick, Limerick, Ireland; 3 Irish World Academy of Music and Dance, University of Limerick, Limerick, Ireland; 4 Research Centre for Arts and Wellbeing, Edge Hill University, Ormskirk, United Kingdom; 5 School of Nursing and Midwifery, University of Limerick, Limerick, Ireland; 6 School of Medicine, University of Limerick, Limerick, Ireland

**Keywords:** antenatal music therapy, intervention development, perinatal mental health, TIDieR checklist, women’s health

## Abstract

**Introduction:**

Perinatal mental health can significantly impact parental and infant wellbeing. Music interventions have shown promise in improving perinatal wellbeing during pregnancy. However, research in this area remains scarce, and very few structured music therapy programmes have been developed and reported in sufficient detail to enable replication and implementation in clinical settings. This article aims to describe the development process of a novel Specialised Antenatal Music Therapy (SAMT) intervention and provide a detailed report of this using the Template for Intervention Description and Replication (TIDieR) 12-point checklist.

**Methods:**

Guided by the Medical Research Council (MRC) framework for developing complex interventions, SAMT was developed through a systematic, evidence-based, theory-driven, and stakeholder-informed process. This included: (1) assembling a multidisciplinary expert team, (2) reviewing existing research evidence and antenatal music therapy protocols, (3) understanding the context and engaging stakeholders through research and co-creation, (4) drawing on existing theories and techniques to facilitate treatment outcomes, and (5) evaluating the feasibility and efficacy through a proof-of-concept study.

**Results:**

This systematic approach resulted in the development of SAMT - a novel music therapy programme for pregnant women attending a Specialist Perinatal Mental Health Service (SPMHS) in Ireland. SAMT consists of six weekly individual music therapy sessions, each lasting 45–60 minutes, delivered either online or in-person at the SPMHS outpatient clinic in a central location. Key components include music-assisted relaxation, identifying a bonding song, musical bonding, song discussion, songwriting, creating pregnancy/birth playlists, and creative music making. Early feasibility testing indicated that the intervention was acceptable and well received by participants.

**Discussion/conclusion:**

Using the TIDieR checklist to report complex interventions provides a structured, systematic way of conveying necessary detail. This method enables music therapists, clinicians, and researchers to understand the SAMT’s underlying rationale, theoretical foundation, delivery methods, and implementation considerations, facilitating replication, refinement, and integration into perinatal mental health services and research.

## 1 Introduction

Pregnancy represents a profound life transition, characterised by significant physical, psychological, and social changes, including bodily transformations, hormonal fluctuations, and shifts in identity, relationships, and career roles (1). Perinatal mental health refers to the psychological and emotional wellbeing of women during pregnancy and the first year after birth. A healthy transition to parenthood requires adapting to changes in ways that foster positive coping strategies, emotional regulation, resilience, meaning-making, and secure attachments with the infant, while preserving a strong sense of identity and agency (2).

While this period can be transformative and empowering, it may also involve psychological challenges that require sensitive, woman-centred support. Factors such as financial stress, social isolation, relationship difficulties, intimate partner violence, prior mental illness, discontinuation of psychotropic medication, previous pregnancy loss, traumatic birth experiences, and other adverse life events can heighten vulnerability for some women (1, 3, 4). Research reveals up to 27% of women experience mental health difficulties in early pregnancy, with anxiety and depression being most common (5). Globally, up to 25% and 17% of pregnant women report symptoms of anxiety (6) and depression respectively (7). If left unaddressed, perinatal mental health conditions can increase the risk of postnatal depression and anxiety, impair maternal functioning, and diminish quality of life (7). They are among the leading causes of maternal mortality, with suicide being the most common direct cause of death during the perinatal period in the UK and Ireland (5). These conditions are also associated with difficulties in mother-infant bonding, which may affect long-term emotional and behavioural development in children (8). Systematic reviews show that depression and anxiety during pregnancy significantly increase the risk of adverse birth outcomes, including up to a 140% higher risk of preterm birth and a 60-80% higher risk of low birth weight (9–12). These outcomes may result from elevated maternal stress hormones, such as cortisol and catecholamines, which affect placental function, foetal growth, and uterine blood flow (13, 14). Maternal stress during pregnancy has also been shown to influence foetal brain development and is associated with increased risks of neurodevelopmental and health problems in early childhood, including ADHD, ASD, emotional difficulties, cognitive impairments, and asthma (13, 15, 16), as well as anxiety disorders and internalising behaviours in adolescence (17, 18).

Perinatal mental health problems impose a significant economic burden, with lifetime costs per annual birth cohort estimated at £8.1 billion in the UK (19), $14.2 billion in the United States (20), $7.3 billion in Australia (21), $7.3 billion in Brazil (22), and $2.8 billion in South Africa (23). In response, there is a growing emphasis on strategies that nurture women’s emotional wellbeing, strengthen their capacity for self-care and resilience during pregnancy, and foster a positive, empowering transition to parenthood (24).

Music-based interventions have gained recognition as effective non-pharmacological approaches to support emotional regulation, reduce stress, and foster maternal-foetal bonding during pregnancy (25). These interventions range from passive listening to recorded music to active engagement activities such as lullaby programmes and singing groups (26). Music therapy is distinct in being delivered by a credentialed therapist within a therapeutic relationship, using personalised and interactive musical experiences tailored to individual needs. This live, responsive process offers a non-invasive and engaging option for promoting emotional well-being and addressing mental health challenges. Its adaptability, low stigma, and non-invasive nature make it an appealing alternative to conventional talk therapy and pharmacological treatments during pregnancy (27).

To the authors’ knowledge, there is no published protocols or guidelines specifically tailored for antenatal music therapy aimed at women experiencing mental health conditions. It is crucial to provide substantial information about the theoretical basis, the components and the operational definitions of an intervention to promote fidelity and maximise the likelihood of intervention effectiveness (28). A comprehensive published description of an intervention can assist researchers, clinicians, and policy makers to replicate or build on research findings and to implement effective, sustainable interventions, thereby reducing research waste (29). This article aims to describe the development of a novel Specialised Antenatal Music Therapy (SAMT) intervention for pregnant women experiencing mental health conditions, guided by the Medical Research Council (MRC) framework for complex interventions and reported using the Template for Intervention Description and Replication (TIDieR) 12-point checklist (29) to enhance transparency, optimise impact, and improve accessibility of antenatal music therapy.

## 2 Methods

Guided by the MRC framework for developing and evaluating complex interventions, SAMT was developed through a systematic, evidence-based, theory-driven, and stakeholder-informed process. This included:

1. assembling a multidisciplinary expert team,
2. reviewing existing research evidence and antenatal music therapy protocols,
3. understanding the context through a cross-sectional study with stakeholders including expectant and new mothers, their partners, and perinatal healthcare professionals,
4. drawing on existing theories and techniques to facilitate treatment outcomes, and
5. evaluating the feasibility and efficacy.

Each of these steps is discussed in detail in the following sections.

### 2.1 Assembling a multidisciplinary expert team

One of the key actions in intervention development is the formation of a team with relevant expertise (30). The multidisciplinary research team supporting the development of SAMT comprised two credentialed music therapists (Author 1 & 4) with academic backgrounds in psychology and music alongside extensive clinical practice experience in mental health, one of whom is also an Associate Professor (Author 4) with a strong track-record in participatory health research and music therapy service development. The team also included an Associate Professor of Nursing and Midwifery (Author 2) with clinical practice experience in maternity, and community care in addition to research expertise in perinatal, maternal and child mental health, and the delivery of services to marginalised and socially excluded populations. The final team included a consultant psychiatrist (Author 3) and the clinical lead of the regional perinatal mental health service in Ireland, where the intervention was developed.

The music therapist (Author 1) who led on the development and delivery of the intervention also received international advanced training in specialised perinatal music therapy techniques, including Music Therapy Assisted Childbirth (MTACB), birth music consultation, prenatal music bonding, NICU music therapy, and creative arts birthing designed to support the well-being of families throughout the prenatal, childbirth, and postnatal journey.

International experts in perinatal music therapy were also consulted during the intervention development stage. These included notable music therapists based in the United States and the United Kingdom, who were clinical leads in their position and held extensive experience working with pregnant women and new parents across diverse clinical and cultural contexts. Their contributions helped ensure the intervention was theoretically grounded, culturally sensitive, and clinically relevant.

### 2.2 Reviewing existing research evidence and antenatal music therapy protocols

The MRC guidance proposes that the optimal approach to developing interventions is to apply the best available evidence (31). To inform the development of the intervention, a review of key literature on antenatal music interventions was undertaken, both within and outside the specialist field of music therapy. This included a synthesis of documented approaches to antenatal music therapy, focusing on their structure, delivery, and reported outcomes.

#### 2.2.1 Research evidence on music and perinatal mental health during pregnancy

A substantial body of evidence indicates that music interventions during pregnancy are associated with a decrease in maternal anxiety (25). A meta-analysis analysed five studies involving 1261 pregnant women and reported that music interventions significantly reduced levels of maternal anxiety (SMD = -0.21, 95% CI: -0.39 to -0.03) (32). A similar effect (SMD=-0.42, 95% CI: -0.47 to -0.08) was reported from another meta-analysis of 11 studies with 1482 participants and noted the significant anxiety-reducing effects of music listening at home (33). Additionally, research indicates that listening to relaxing or preferred music can effectively decrease anxiety in women awaiting amniocentesis (34) and during transvaginal ultrasound (35). Besides anxiolytic benefits, music interventions have been shown to reduce blood pressure and pregnancy-induced hypertension (36), improve sleep quality in pregnant women with disturbed sleep (37), improve lower back pain and increase quality of life during pregnancy (38). The specialist practice of music therapy has also been shown to significantly reduce pregnancy-related distress for women with high-risk pregnancy experiencing extended antepartum hospitalisation (39, 40).

Evidence also suggests that the positive effect of antenatal music engagement can continue beyond pregnancy. For maternal wellbeing and childbirth experience, a RCT found that music listening during pregnancy was associated with a more positive birth experience, characterised by shorter labour duration, higher likelihood of natural onset of delivery, and reduced need for medication (41). Postnatally, listening to music and singing during pregnancy were associated with higher levels of postnatal maternal wellbeing and lower symptoms of postnatal depression during the first three months after birth (42).

In terms of neonatal outcomes, singing lullabies during pregnancy was linked to stronger postnatal attachment between mother and infant, and a decrease in neonatal crying episodes, infantile colic, and nightly awakenings (43). Infants whose mothers listened to music during pregnancy scored better on the Neonatal Behavioural Assessment Scale (NBAS), indicating enhanced neonatal behaviours (44). Moreover, antenatal music exposure led to long-term neural effects in infants, suggesting that the benefits of such exposure extend well beyond birth (45). This is supported by a recent systematic review that found antenatal sound stimulation, including music, can form stimulus-specific memory traces during the foetal period and affect neonatal neural systems (46). Lastly, a recent study found that antenatal music exposure may facilitate early language processing and acquisition (47). These findings suggest that antenatal music engagement not only benefits maternal mental health but also has a profound impact on infant development and behaviour, with effects that may persist well into childhood.

#### 2.2.2 An overview of antenatal music therapy practice

A targeted literature search identified 10 records of antenatal music therapy practice in English. This section provides an overview of these specialised interventions, which differ from other music-based intervention by involving a qualified music therapist who uses specific music techniques tailored to the unique needs and experiences of pregnant women, their foetuses, and families within a therapeutic relationship. Of the 10 records, five focused on bedside music therapy (39, 40, 48–50), three described group programmes (51–53), one reported home/family-based therapy (54), and one addressed perinatal palliative music therapy (55). These programmes are discussed in detail below and summarised in **Table 1**.

**Table 1:** A synthesis of antenatal music therapy programmes described in the literature.

Author	Article type	Setting	Duration	Intervention aims	Intervention components	Target group	Outcomes	Country
Winslow (1986) (50)	Case Study	Hospital-based/ bedside	Individual 20 to 30-min session and group session	To alleviate anxiety regarding pregnancy and labour outcome	Individual: Learning and practicing of progressive relaxation techniques with music and guided imagery, verbal processing, talking to the foetus Group: lullabies sing-along and group discussion	Individual: Hospitalised antepartum women Group: pregnant women and new mothers with their babies	Reduction of anxiety, somatic complaints; increased level of compliance with treatment; increased personal awareness of using music as coping mechanisms	USA
Federico (2005) (53)	Conference presentation	Group	Unspecified	To strengthen early parents-baby bonding; to improve pregnancy and birth experience; to improve baby’s quality of life	Relaxation through movement, creative visualisation with music, welcoming song, sound bath, vibrational massage, improvisation with musical instruments, prenatal music stimulation	Pregnant women (wide range of conditions)	N/A	Argentina
Friedman et al. (2010) (52)	Programme description and evaluation	Group	Weekly 1-hour sessions for 6–8 weeks	For mothers to use music for self-expression, coping and inspiration, and calm babies.	Music-assisted relaxation, music listening, lyrics analysis, interactive singing and instrument playing, songwriting, personalisation of precomposed lullabies	Pregnant women and new mothers; teen parents	Mother feeling more relaxed, less anxious, more able to care for their babies, able to use activities at home.	USA
Bauer et al. (2010) (40)	RCT	Bedside	One 1-hour session	To alleviate antepartum-related distress	Music-facilitated relaxation, active music listening, songwriting, music for bonding, clinical improvisation	Hospitalised pregnant women	Significant reduction of antepartum distress (ABEII)	USA
Lander (2017) (54)	Case study	Family-Based	1–4 antenatal and postnatal session	For parents to explore and connect with their unborn child, building resilience and confidence	Using melody, rhythm, and voice to promote awareness of self and the foetus, support bonding, encourage communication between parents and foetus	At risk expectant mothers and fathers (history of addition and other psychosocial needs)	Improved confidence in interaction with babies, increased awareness around positive behaviours and motivation to attend local groups & antenatal appointments	Scotland
Schreck & Economos (2018) (55)	Case study	Family-Based Palliative Music Therapy	Unspecified	To provide comfort to the families, create a lasting memory, and help in the bereavement process	Recording heartbeats of foetuses and parents, incorporating into a co-created song	Expecting parents facing terminal diagnosis for their foetus	Strengthened connections with loved ones, creating meaningful shared experiences, serving as a catalyst, support bereavement	USA
Tecken-berg-Jansson et al. (2019) (49)	RCT	Bedside	Three daily 30-min sessions for three consecutive days	To relieve stress and anxiety	Vibroacoustic intervention using lyre instruments (a Swedish 7-string pentatonic lyre and German Tao-lyre), varying decibel levels, humming lullabies	Hospitalised pregnant women	An increase in the SD2 measure during music therapy sessions, suggesting relaxation effects	Finland
Corey et al. (2019) (48)	Mixed methods retrospect analysis	Bedside	One 30-min session	To alleviate stress and anxiety, provide emotional support	Personal relaxation, receptive music listening, music-assisted relaxation, breath entrainment, humming, toning, guided imagery, mindfulness exercise, body scan, verbal validation and support	Hospitalised antepartum women (including postpartum women and their infants)	High satisfaction, positive effects on relaxation and sense of connection with baby.	USA
Carvalho et al. (2021) (51)	Longitudinal and microanalytic case study	Group	12 weekly 90-minute sessions	To promote prenatal bonding	Group musical improvisation, humming and singing, creating songs using “musical parody” technique, creating a welcome song with family members	Pregnant women	Foetal motor self-exploration, longer infant vocalisations during prenatal song, increased pitch in maternal singing	Portugal
Horn et al. (2022) (39)	Mixed methods	Bedside	One to five 50-min sessions	To improve emotional well-being	Guided relaxation, lyric analysis and discussion, song dedication, songwriting, creative music making	Hospitalised pregnant women (including family sometimes)	Improved connections to family, normalisation of the hospital experience, emotional relief, and new resources to manage the hospital experience	USA

##### 2.2.2.1 Bedside music therapy

Bedside music therapy for hospitalised pregnant women emerged as the most frequently reported type of antenatal music therapy in the literature. This included one case study (50), two RCTs (40, 49), and two mixed methods studies (39, 48). Winslow’s (1986) groundbreaking work documented both group and individual music therapy for pregnant women who were hospitalised due to medically deemed high-risk pregnancies (50). Individual sessions of 20–30 minutes duration, three to four times per week, focussed on teaching and practicing progressive relaxation techniques accompanied by music and guided imagery, verbal processing and open discussion with the foetus. A weekly group session of singing lullabies and children’s songs was also offered for pregnant women, new mothers and their babies, providing a space to foster group discussions on experiences of hospitalisation, labour, and care for newborns.

Three studies have described a bedside intervention that incorporated a range of music therapy techniques. These include music-facilitated relaxation, active music listening, songwriting, song discussion, music for bonding and clinical improvisation (39, 40, 48). Bauer et al. (40) and Corey et al. (48) both focused on single bedside sessions lasting 30–60 minutes while in the study of Horn et al. (39)women received up to five sessions with scope to also include their families if women so wished. Lastly, Teckenberg-Jansson et al. (49) detailed a specific vibroacoustic intervention for relaxation using two lyres (stringed musical instruments), played at varying decibel levels (ranging from 10 to 35 dB) according to each pregnant woman’s level of comfort. During the intervention, the music therapist placed and played the lyres on the participating women’s abdomen, legs, and back as they lay comfortably on their bed while being encouraged by the therapist to hum lullabies.

##### 2.2.2.2 Group music therapy programme

Three publications identified through the targeted search described group-based antenatal music therapy interventions. These included two programme descriptions - *Focal Music Therapy for Obstetrics (FMTO)* (53) and *Lullaby 101* (52) – and a case study exploring a woman’s experience within a 12-week songwriting music therapy group (51).

*FMTO* was designed to strengthen parents-foetal bonding and improve maternal and infant wellbeing (53). The programme incorporated techniques such as relaxation through movement, creative visualisation with music, a welcome song, sound bath, vibrational massage, musical improvisation (the spontaneous creation of music on musical instruments), and prenatal music stimulation (using music and sound to stimulate the foetus).

*Lullaby 101* was a 6-8-week programme for pregnant women and new mothers with mental health conditions including depression, bipolar disorder, or psychosis (52). This programme involved techniques such as music-assisted relaxation, music listening, lyrics analysis, interactive singing and instrument playing, songwriting, and personalisation of precomposed lullabies. It adopted a curriculum-based model covering topics such as music use in daily life, lullaby composition, identifying signs of infant distress, and choosing calming music.

Carvalho et al. (51) conducted a longitudinal, micro-analytic case study of a mother–infant dyad, tracking interactions from late pregnancy through three months postpartum of a woman who attended a 12-week songwriting group intervention, where five pregnant women collectively created songs for their unborn infants. The process included group musical improvisation, humming and singing, and composing new songs using a musical parody technique, in which pre-existing lyrics were adapted to make them personally meaningful. The group also co-created a welcome song for each unborn baby, with the option for family members to participate if desired. In the micro-analytic follow-up of the mother-infant dyad at nine days and three months postpartum, it was observed that infant vocalisations were significantly longer when hearing the prenatal song compared with humming or silence, and that the mother adapted her singing by increasing pitch following the infant’s vocalisations. These findings suggest that an original prenatal song may foster reciprocal musical attunement and enhance early mother-infant interaction.

##### 2.2.2.3 Home/family-based music therapy

Two publications identified through the targeted search described unique family-based music therapy programmes: the *BabySounds* project (54) and *Heartbeat Recording Palliative Music Therapy* (55).

The *BabySounds* project was a one-year initiative designed for psychosocially vulnerable first-time expectant and new parents who might have a history of addiction, violence, trauma, and abuse. It was aimed at supporting positive parenting, social and emotional health, and social inclusion, and consisted of up to four home visit sessions with pregnant women and their partners during the antenatal and postnatal period. The process involved using melody, rhythm, and voice to promote awareness of self and the foetus, support bonding, and encourage communication between the parents and their foetus.

Schreck and Economos (55) described a music therapy approach for expectant parents whose foetus was given a terminal diagnosis. The unique intervention involved capturing internal body sounds such as the heartbeats of the foetus and the parents using Doppler and other recording devices. These recordings were then integrated into a song co-created by the music therapist and the parents. This musical creation served as both a therapeutic outlet and a tangible means for parents and families to process and express grief.

##### 2.2.2.4 Pregnancy-related music therapy techniques

In most of these music therapy programmes, a diverse array of music therapy techniques was offered and often employed in combination. Beyond traditional music therapy techniques, specialised techniques have been devised to meet the unique needs of pregnant women. These include creating birthing playlists (56), singing and personalising lullabies (52), writing a womb song or a welcome song for the infant (51, 57), vibroacoustic stimulation through lyre instruments (49) and composing with the recording of parents’ and foetal heartbeats (55, 58).

Qualitative findings from these studies and programme reports reveal that music therapy supported maternal wellbeing by offering a space to relax and process thoughts (48), providing a medium for emotion processing and release (39, 52), normalising and enhancing the hospital experience, and equipping women with new resources (39, 48). Beyond the individual level, it also helped foster mother-foetal bonding, connect the family, celebrating life, and create lasting legacies and memories (39, 54, 55, 57).

### 2.3 Understanding the context and engaging stakeholders through research and co-creation

In line with the MRC Framework for Developing and Evaluating Complex Interventions, the design of SAMT was shaped by a comprehensive understanding of the local context, including service structures, policy frameworks, and stakeholder perspectives. These principles informed the intervention’s design to enhance its feasibility, acceptability, and potential for long-term implementation (30, 31). Additionally, SAMT was co-created and refined with participating pregnant women, ensuring that its content and delivery were meaningful, relevant, and adaptable to their needs and experiences.

#### 2.3.1 The Irish context

Perinatal mental health challenges in Ireland mirror global trends, with approximately one in five women experiencing mental health conditions during the perinatal period, with one in seven reporting moderate to severe depression, and one in ten reporting moderate to severe anxiety (59). A recent survey in Ireland indicated that certain indices of sociodemographic and socioeconomic disadvantages such as being a young, single or first-time mother, lower education background, unemployment, or having relationship difficulties, increased the likelihood of poor mental health during the perinatal period (59).

In response to these challenges, the Department of Health in Ireland published the first *National Maternity Strategy 2016–2026* to advocate for a holistic, women-centred, and biopsychosocial model of care, aiming to reduce the medicalisation of pregnancy and childbirth by promoting preventive measures and early intervention, particularly in relation to mental health (60, 61). Complementing mental health policies, *Sharing the Vision: A Mental Health Policy for Everyone* (61) emphasises the need for a comprehensive, multidisciplinary approach that considers biological, psychological, and social factors contributing to mental illness.

Building on these strategic frameworks, Ireland launched *the Specialist Perinatal Mental Health: Model of Care* in 2017 (Health Service Executive, 2017) (62). This initiative led to the establishment of Specialist Perinatal Mental Health Services (SPMHS) across six major hospital groups, including the National Maternity Hospital, Rotunda Hospital, Coombe Women & Infants University Maternity Hospital, University Maternity Hospital Limerick, Cork University Maternity Hospital, and Galway University Hospital. These services support women experiencing new onset, relapse, or recurrence of mental health disorders during the perinatal period. Each SPMHS team is led by a consultant psychiatrist in perinatal psychiatry and includes a multidisciplinary team comprising a non-consultant hospital doctor, advanced midwife practitioner, mental health nurse, senior clinical psychologist, mental health social worker, and occupational therapist. The primary focus is on the early support, assessment, and treatment of perinatal mental health disorders. Referrals to the service are accepted from healthcare professionals such as general practitioners, obstetricians, public health nurses, and midwives for various mental health difficulties, primarily perinatal mood and anxiety disorders (PMADs), including pregnancy-related anxiety and tokophobia.

Despite these advancements in awareness and service provision (5), recent studies (59, 63, 64) indicate that perinatal mental health services in Ireland remain insufficient. The surge in referrals to SPMHS has led to prioritisation of women with more severe symptoms, potentially delaying care for those with milder presentations (65). Additionally, stigma and shame surrounding mental health diagnoses often deter women from seeking help (66). Many avoid or discontinue psychotropic medication during pregnancy or breastfeeding due to concerns about potential risks, expressing a preference for non-pharmacological interventions (66). These findings underscore the pressing need for holistic, accessible, and effective treatment options for women requiring additional mental health support during the perinatal period.

#### 2.3.2 Stakeholder perspectives and preferences

The MRC framework highlights that stakeholder involvement is critical to ensuring an intervention is contextually relevant and adaptable, responsive to the needs, values, and contexts of those they are intended to serve. To achieve this, a mixed-methods cross-sectional study named *Music and Perinatal Wellbeing* was carried out to explore stakeholders’ experiences and perspectives on music and music therapy in perinatal care. In the context of SAMT, key stakeholders are maternity service users, including both parents, and providers such as perinatal healthcare professionals and other perinatal care providers in Ireland. The *Music and Perinatal Wellbeing* study consisted of online surveys with 254 women and 11 partners, and 46 healthcare practitioners and follow-up interviews with six stakeholders recruited from the survey respondents (67–69).

Findings revealed widespread recognition of music’s benefits during pregnancy and childbirth, including support for relaxation, mood regulation, sleep, energy, bonding, labour, and the clinical environment. However, many respondents noted a lack of guidance on how to use music effectively during the perinatal period and expressed strong interest in the integration of music therapy into standard maternity care. Preferred programme features included support for relaxation, parent-infant bonding, and childbirth preparation (67, 68).

Semi-structured interviews with two new mothers and four perinatal healthcare professionals further identified practical considerations for implementation. Stakeholders emphasised the importance of safe, holistic, and accessible support for women experiencing psychological distress, particularly when this affects bonding or maternal self-care. The third trimester and hospital admission were highlighted as key windows for intervention. Additionally, stakeholders stressed the need for structured guidelines and public awareness to support sustainable integration of music therapy into maternity care (69).

#### 2.3.3 Co-creating and refining the intervention with patients

Building on the insights gained from stakeholder engagement and contextual analysis, the next critical step in the development of SAMT was co-creating the intervention directly with the women it aimed to support. This ensured that the design was not only informed by broader policy and service structures but also deeply attuned to individual experiences and preferences.

Co-creation involves working directly with end-users to tailor interventions to specific contexts, ensuring that the content and delivery are meaningful, relevant, and adaptable. This approach has been increasingly applied in health intervention development and has been shown to enhance both effectiveness and sustainability (70).

In the development of SAMT, co-creation was embedded in the therapeutic process. Pregnant participants were actively involved in shaping the intervention through co-designing the session structure, and the selection and creation of personalised music resources, including playlists and original songs that reflected their emotional and relational experiences. Session structures were flexibly adapted to accommodate individual preferences, evolving needs, and feedback over time. This dynamic and responsive approach allowed participants to engage meaningfully in the intervention and to influence its direction.

Importantly, co-creation fostered a sense of ownership among participants, which emerges through an empowerment process that supports openness, shared decision-making, and perceived control (70). By involving participants in the design and delivery of SAMT, the intervention was not only grounded in evidence and clinical expertise but also enriched by the voices and values of those it aimed to support.

### 2.4 Drawing on existing theories and techniques to facilitate treatment outcomes

In line with the MRC guidance on complex intervention development, the development of SAMT was informed by existing theories that illuminate the psychological, social, and relational processes shaping maternal wellbeing and antenatal bonding. Four interrelated frameworks guided the therapeutic approach: salutogenesis and resource-oriented music therapy; humanistic person-centred theory; self-determination theory (SDT); and attachment theory, including concepts of communicative musicality and affect attunement.

#### 2.4.1 Salutogenesis theory, matrescence, and resource-oriented music therapy

The concept of salutogenesis, as introduced by Antonovsky in the late 1970s, focuses on individuals’ resources and capacity to create health rather than the focus on risks and disease (71). Complementing this, the concept of matrescence recognises the transition to motherhood as a normative developmental process involving significant physical, emotional, and social change rather than a condition or disease to be treated (72). This shift highlights the importance of resilience, adaptation, and the mobilisation of internal and external resources (73).

Within this framework, the resource-oriented music therapy approach is adopted as the theoretical foundation for SAMT. Resource-oriented music therapy is a therapeutic approach that focuses on identifying and utilising the inherent resources and strengths of individuals rather than solely addressing deficits or problems (74). Individual preferences, experiences and abilities are emphasised. The music therapist’s role is to guide and support individuals in their musical journey and help them to recognise and utilise their musical resources. By focusing on strengths rather than limitation, resource-oriented music therapy promotes empowerment, self-awareness, and resilience. By leveraging music as a resource, music therapists can help pregnant women develop a sense of coherence and access generalised resistance resources to cope with the challenges from pregnancy onward. Mastnak (75) proposes that antenatal music therapy can achieve these through four focuses: 1. management of antenatal stress, anxiety, and depression through music; 2, mental and physical birth preparation through cognitive adjustment, emotional regulation, physical activity, relaxation, pain management, and social inclusion; 3. music-associated bonding and self-efficacy; and 4. acoustic stimulation to support foetal development.

#### 2.4.2 Humanistic person-centred and self-determination theory

Another theoretical framework that underpins SAMT is humanistic person-centred music therapy that is built on Carl Roger’s (76) humanistic person-centred theory and SDT (77). The humanistic person-centred approach is rooted in the belief that each individual possesses inherent worth and the capacity for growth and self-actualisation. Drawing on Carl Roger’s person-centred therapy, the therapeutic relationship and environment are characterised by empathy (experiencing life from the client’s perspective), unconditional positive regard (nonjudgemental and respectful acceptance), and congruence (transparency and trustworthiness). In humanistic music therapy (78), the psychotherapeutic space can facilitate “personal and transpersonal development of the person through sound and music is facilitated, using an approach emphasising respect, acceptance, empathy and congruence”. In the context of SAMT, these principles inform the therapeutic orientation of the music therapist who facilitates a supportive, safe and respectful space for musical and psychological exploration. Sessions are tailored to each woman’s unique emotional experiences, cultural background, and personal preferences, allowing music to become a medium for self-expression, emotional processing, and connection. The “music” in SAMT is considered an embodiment of human agency and a resource in the form of social capital, through which individuals can work toward self-actualisation (79).

In addition, SAMT supports the basic psychological needs outlined in the SDT. SDT posits that wellbeing is fostered when three basic psychological needs are met: autonomy, competence, and relatedness (77). To support these needs, the person-centred, co-creative approach of SAMT allows individuals to choose which health needs they wish to address in each session and how they engage with music through an open, adaptable session structure. This freedom promotes autonomy by giving individuals control over their therapeutic experience, which is particularly important in mental health contexts and for those affected by trauma. In SAMT, competence may be reflected in women feeling empowered to express themselves, engage with their foetus through musical experiences, and experience a sense of accomplishment from singing, playing, or composing music for their baby. Relatedness is nurtured through shared musical experiences, attuned listening, and co-creation with both the music therapist and the foetus.

#### 2.4.3 Attachment theory, communicative musicality and affect attunement

Attachment theory, first proposed by Bowlby (80), describes the dynamic and reciprocal relationship between the primary caregivers and their infant, with the aims to seek proximity and provide safe, security, protection, and affect regulation to the child (81, 82). Secure attachment develops when caregivers respond promptly and consistently to an infant’s distress signals, such as crying or seeking proximity, creating a reliable sense of safety that supports socio-emotional development throughout life (80, 81). When these early bonds are disrupted due to factors such as maternal mental health difficulties, trauma, high-risk pregnancy, or social stressors, infants often develop insecure patterns such as avoidant (characterised by emotional withdrawal and suppression of attachment needs), ambivalent (marked by heightened anxiety and preoccupation with caregiver availability), or disorganised (involving contradictory, fear-based behaviours) attachment as adaptive responses to inconsistent, unavailable, or frightening caregiving (81, 83).

Many scholars propose that the attachment bond begins during pregnancy, as the foetus is perceived as an individual with its own identity and distinct needs (84). The concept of “mother-foetal attachment” was first introduced by Cranley (85), who described it as “the extent to which women engage in behaviours that represent an affiliation and interaction with their unborn child” (p. 282). Muller (82)expanded on this by highlighting the role of preparedness, fantasies, affection, and interaction. Antenatal attachment is characterised by the parents’ emotional states, cognitive processes, and behavioural patterns directed towards the foetus, including activities such as naming the foetus, engaging in interaction, initiating communication, tenderly touching the abdomen, providing care, and undertaking preparations for the forthcoming arrival of the newborn. Strengthening this bond during pregnancy has been shown to positively influence postnatal attachment and reduce the risk of bonding disorders (84).

Communicative musicality, as described by Malloch and Trevarthen (86), refers to the innate musical qualities of human interaction that support emotional connection through rhythm, timing, and expressive contour. *Affect attunement*, introduced by Daniel Stern (87), describes how caregivers synchronise their emotional expressions and responses with those of infants, fostering emotional connection and attunement. These two concepts originally applied to the interaction between an infant and the caregiver after birth. Emerging evidence that foetuses respond to auditory stimuli from around 16 weeks’ gestation (88) suggests that these communicative processes may begin before birth, providing a foundation for early affective attunement through sound (89). In SAMT, singing and musical engagement are used to foster antenatal attachment by creating opportunities for emotional connection and rhythmic interaction. Maternal singing serves as a natural medium for soothing, bonding, and shared emotional expression (90). The soothing melodies and rhythmic patterns of music can help regulate the foetus emotional state and promote feelings of security and comfort, supporting positive emotional bonding even before birth (91). These theories and findings underscore the potential of SAMT during pregnancy on the formation and enrichment of the mother-foetal relationship, highlighting its important role in early intervention to prevent and repair disrupted attachment (92).

### 2.5 Evaluating feasibility and efficacy through a proof-of-concept study

The MRC Framework for Developing and Evaluating Complex Interventions emphasises the importance of iterative testing to assess real-world applicability. In line with the guidance, the next phase of intervention development involved assessing the feasibility and preliminary efficacy of SAMT through a proof-of-concept (PoC) study. The full empirical findings of the SAMT PoC study have been published elsewhere (27) and a concise summary is provided here to support the methodological focus of this report.

This PoC study employed a convergent mixed methods design with pregnant women attending the SPMHS in Ireland. Quantitative data included pre- and post-intervention self-rated outcome (SRO) measures of mental wellbeing (SWEMWBS), anxiety (GAD-7), and antenatal attachment (PAI), collected at the beginning and after completion of the six SAMT sessions. Session-level ratings of anxiety and perceived closeness to the baby were recorded through Visual Analogue Scale (VAS) before and after each session. All SRO measures were self-administered by participants via online questionnaires accessed through links provided prior to each assessment point. Additionally, feasibility indicators including intervention adherence and fidelity were recorded. Further details on the feasibility indicators are reported in the Results section (3.11). Qualitative data were gathered through self-administered post-programme questionnaires and semi-structured interviews, which were conducted by an independent researcher not involved in the intervention to minimise bias.

Women with mental health conditions were referred to SAMT by the psychiatrist, midwife, nurse, and social worker at SPMHS. SAMT sessions were delivered by the lead researcher, who was a music therapist at SPMHS; therefore, therapist blinding was not feasible. A total of 12 women were enrolled, nine participants (mean age: 29 years; range: 23-36) completed SAMT and pre/post online questionnaires, and seven participated in semi-structured interviews. Findings indicated that SAMT was effective, feasible and well-received, with a 75% retention rate and high adherence (see 3.12 for further details). Statistically significant improvements with large effect sizes were observed in wellbeing (SWEMWBS: mean increased from 19.36 to 23.37; *p* < 0.001; *d* = −1.77), anxiety reduction (GAD-7: mean decreased from 10.33 to 4.11; *p* < 0.001; *d* = 2.32), and antenatal attachment (PAI: median increased from 56 to 69; *p* = 0.007; *r* = −0.89). Importantly, every individual participant showed positive improvement exceeding the minimal clinically important difference (MCID) across these outcomes regardless of delivery modes or component selection. Immediate reductions in anxiety and increased closeness to their baby were also observed after each session (VAS-anxiety: median decreased from 5 to 2; VAS-closeness: median increased from 7 to 8; both p <.001; r = −0.77), with effects increased progressively over the course of six sessions (27).

Qualitative feedback underscored the acceptability and therapeutic value of SAMT. Participants particularly valued the person-centred and co-creative approach, as some initially expressed apprehension about engaging in activities outside their comfort zone but felt reassured that the sessions were tailored to their individual needs and preferences. Many highlighted its positive impact on emotional regulation, bonding, and self-care. Although participants reported challenges such as technical issues, difficulty securing private space, and managing emotional responses during online sessions, they appreciated the flexibility of online delivery as crucial for sustaining engagement when faced with mobility limitations and long travel distances.

These findings support the potential of SAMT as a holistic, non-pharmacological intervention for perinatal mental health care, and lay the groundwork for future larger-scale effectiveness and health economic evaluations.

## 3 Results

This section provides a detailed description of SAMT following the TIDieR checklist (29):

Intervention Description (TIDieR Checklist Summary)

### 3.1 Item #1: brief name

Specialised Antenatal Music Therapy (SAMT).

### 3.2 Item #2: Why

SAMT was developed to support emotional wellbeing, maternal–foetal bonding, and anxiety and stress management during pregnancy. While originally designed for women attending specialist perinatal mental health services, the principles of SAMT are applicable to pregnant women more broadly. The intervention offers a non-pharmacological, non-invasive therapeutic option that promotes relaxation, emotional resilience, and a positive connection with the unborn baby.

### 3.3 Item #3: What (materials)

In-Person Sessions

- Private, dedicated therapy room
- Range of musical instruments for expressive and bonding activities
- Audio playback and recording equipment
- Songbooks and lyric sheets
- Guided relaxation scripts and live or recorded relaxation music
- Access to music-streaming services

Online Sessions (In addition to the above)

- Secure video conferencing platform (e.g., Attend Anywhere)
- Laptop or desktop computer with web camera
- Headset with built-in microphone
- Reliable internet connection

Materials for Participants

- Information leaflet with suggestions for home-based relaxation and bonding activities
- Curated relaxation resources or playlists, provided as needed
- Lyric sheets for personalised or collaboratively created songs

### 3.4 Item #4: What (procedures)

SAMT consisted of six weekly individual sessions, each lasting 45–60 minutes. Sessions followed a flexible, person-centred, and co-creative structure that allowed the music therapist to tailor the therapeutic approach to the participant’s emotional state, preferences, and clinical goals at the time of each appointment. The overall flow of each session remained consistent: grounding through breathwork at the start, collaborative selection of session content, and a closing relaxation or bonding exercise. Within this structure, participants exercised full autonomy in choosing among several open or selective components, while core elements—music-assisted relaxation, bonding song identification, and antenatal musical bonding—were incorporated with fidelity across all sessions.

Session 1 focused on orientation to SAMT, exploration of musical history and preferences, clarification of therapeutic goals, and an introduction to relaxation and bonding practices. Sessions 2–5 deepened engagement with core and selective components, with increasing participant autonomy and ongoing negotiation of session pacing and content. From Session 4 onwards, the therapist introduced elements of therapeutic closure, encouraging participants to reflect on progress and consider the goals or creative tasks they wished to complete before the end of the programme. Session 6 served as a consolidation and closure session in which women reviewed their experiences, reflected on personal changes, and identified music-based strategies they intended to continue using in daily life.

The procedures were consistent across in-person, online, and hybrid delivery formats, with the only substantive difference being the availability of musical instruments in the in-person setting. **Table 2** summarises all core and selective components of SAMT, including their therapeutic goals and operational descriptions.

**Table 2:** SAMT therapeutic components, goals, and operational descriptions.

Component type	Component	Goal	Description
Core	Music-assisted relaxation	Reduce anxiety and stress; support emotional regulation; promote self-compassion	Guided relaxation supported by live musical improvisation or therapist-selected recorded music. Themes may include peaceful-place imagery (Session 1), passive relaxation (Sessions 1–6), progressive muscle relaxation (Sessions 2–6), visualisation-based relaxation (Sessions 3–6), birth affirmations (Sessions 4–6), and compassion-focused or loving-kindness sequences (Sessions 4–6).
Core	Identifying a bonding song	Support early attachment; facilitate emotional connection	Collaborative selection of a personally meaningful song (e.g., lullaby, culturally relevant song, or song of kin) used throughout the intervention as the anchor for bonding experiences.
Core	Antenatal musical bonding (receptive and active)	Strengthen maternal–foetal attunement; enhance positive emotion; foster sensory connection	Receptive bonding involves listening to the bonding song or therapist improvisation while visualising closeness, offering loving thoughts, or attending to foetal movement. Active bonding involves singing the bonding song (original, adapted, or vowel-based versions) and/or interactive songs incorporating gentle actions such as stroking, tapping, or rocking to engage with the baby through movement and voice.
Selective	Songwriting or personalising a song	Support emotional expression; integrate pregnancy experiences; enhance agency	Creation of an original song or adaptation of existing lyrics to reflect pregnancy-related thoughts, emotions, values, or hopes (e.g., modifying familiar melodies to express personal experiences).
Selective	Song discussion	Facilitate emotional exploration; process life events	Exploration of emotions, memories, and relational experiences evoked by chosen songs. Discussion may include themes such as grief, attachment, identity, loneliness, family dynamics, or the transition to motherhood.
Selective	Pregnancy or birth playlist creation	Support relaxation; prepare for labour; enhance daily self-care	Joint consideration of musical preferences and emotional responses to selected tracks. Identification of suitable music for childbirth preparation or daily wellbeing (e.g., relaxation, sleep support, emotional regulation).
Selective	Creative singing or music-making	Encourage creativity and emotional release; deepen therapeutic rapport	Vocal improvisation (e.g., toning, two-tone singing, free vocalisation) and/or instrument-based improvisation using percussion or melodic instruments (in-person sessions only).

### 3.5 Item #5: Who provided

SAMT was delivered by an experienced, accredited music therapist with formal perinatal music therapy training and clinical experience in mental health.

### 3.6 Item #6: How

SAMT sessions were delivered individually. Partners or other family members could participate in selected sessions if clinically appropriate and aligned with therapeutic goals.

### 3.7 Item #7: Where

SAMT sessions were delivered either in-person or online based on participant preference. A hybrid option was also available, allowing participants to switch between formats if they were feeling unwell or faced travel difficulties. This flexible model supported continuity of care and responsiveness to individual needs.

#### 3.7.1 In-person setting

The in-person sessions were held in a quiet, accessible outpatient clinic located outside the maternity hospital environment to reduce medicalisation and potential triggering of trauma memories. The clinic provided transport access, parking, toilet and nappy changing facilities, and a private therapeutic space.

#### 3.7.2 Online setting

Participants were encouraged to attend the online sessions at a private, quiet, and comfortable space where they could engage fully in therapeutic activities such as singing, music-making, deep relaxation, and discussion of sensitive matters. To support emotional safety and physical comfort, the following recommendations were provided:

- A supportive armchair with head and arm rests, if available.
- A blanket, water, or a warm beverage for comfort.
- Use of a laptop for optimal audio-visual quality (recommended but not mandatory).
- A stable internet connection to ensure uninterrupted participation.

### 3.8 Item #8: When and how much

SAMT consisted of six weekly sessions, with each lasting 45–60 minutes.

### 3.9 Item #9: tailoring

Tailoring the individual experience was a core principle of SAMT, with a strong emphasis on fostering participant ownership. Each session was adapted to reflect the participant’s emotional state, preferences, and therapeutic goals, ensuring they felt in control, empowered, and actively engaged.

Participants had full autonomy in selecting non-core components, and their agreement was always sought before engaging in core components such as music-assisted relaxation and bonding. Sessions began with open dialogue to identify current needs and preferred activities. For example, a participant who initially focused on bonding chose to explore a family conflict through song discussion and relaxation techniques.

Tailoring extended to the mode of delivery (in-person, online, or hybrid), choice of devices, and session environment. Some online participants found it uncomfortable to engage in creative activities with their camera or microphone on. To support privacy and comfort, they were given the freedom to mute themselves or turn off their camera, whatever helped maximise the therapeutic value of the session.

### 3.10 Item #10: modifications

No modifications or deviations were made to the original design of the SAMT. However, during the course of the proof-of-concept study, the COVID-19 pandemic escalated, leading to restrictions on in-person consultations. As a result, in-person engagement was temporarily suspended, and sessions were conducted exclusively online for a period.

### 3.11 Item #11: How well (planned)

In the proof-of-concept study, SAMT was delivered by the intervention developer hence no additional training was required in this phase. Intervention adherence and fidelity were monitored throughout the SAMT proof-of-concept study as part of the feasibility evaluation. Attendance, delivery mode, and component selection were tracked by the therapist after each session. Participants’ engagement outside of sessions was assessed using a Likert-scale item in the post-intervention questionnaire, capturing their use of music-based strategies and reflections between sessions.

### 3.12 Item #12: How well (actual)

Intervention adherence and fidelity were assessed with nine pregnant participants in the SAMT proof-of-concept study. On average, participants attended 5.33 out of 6 sessions (SD = 1), with missed sessions attributed to hospitalisation, family illness, early delivery, or proximity to due date.

All core components of SAMT (music-assisted relaxation, musical bonding, and bonding song identification) were delivered with 100% fidelity across both online and hybrid formats. Notably, all non-core components were selected by this cohort, with engagement rates ranging from 44% to 89%, reflecting the suitability of all components. Most participants (78%) attended sessions online, while 11% attended in-person only, and 11% transitioned from in-person to online. Post-intervention questionnaire responses indicated frequent use of relaxation resources (mean = 3.67/5) and singing to the baby (mean = 4/5) outside of sessions.

**Figure 1:**
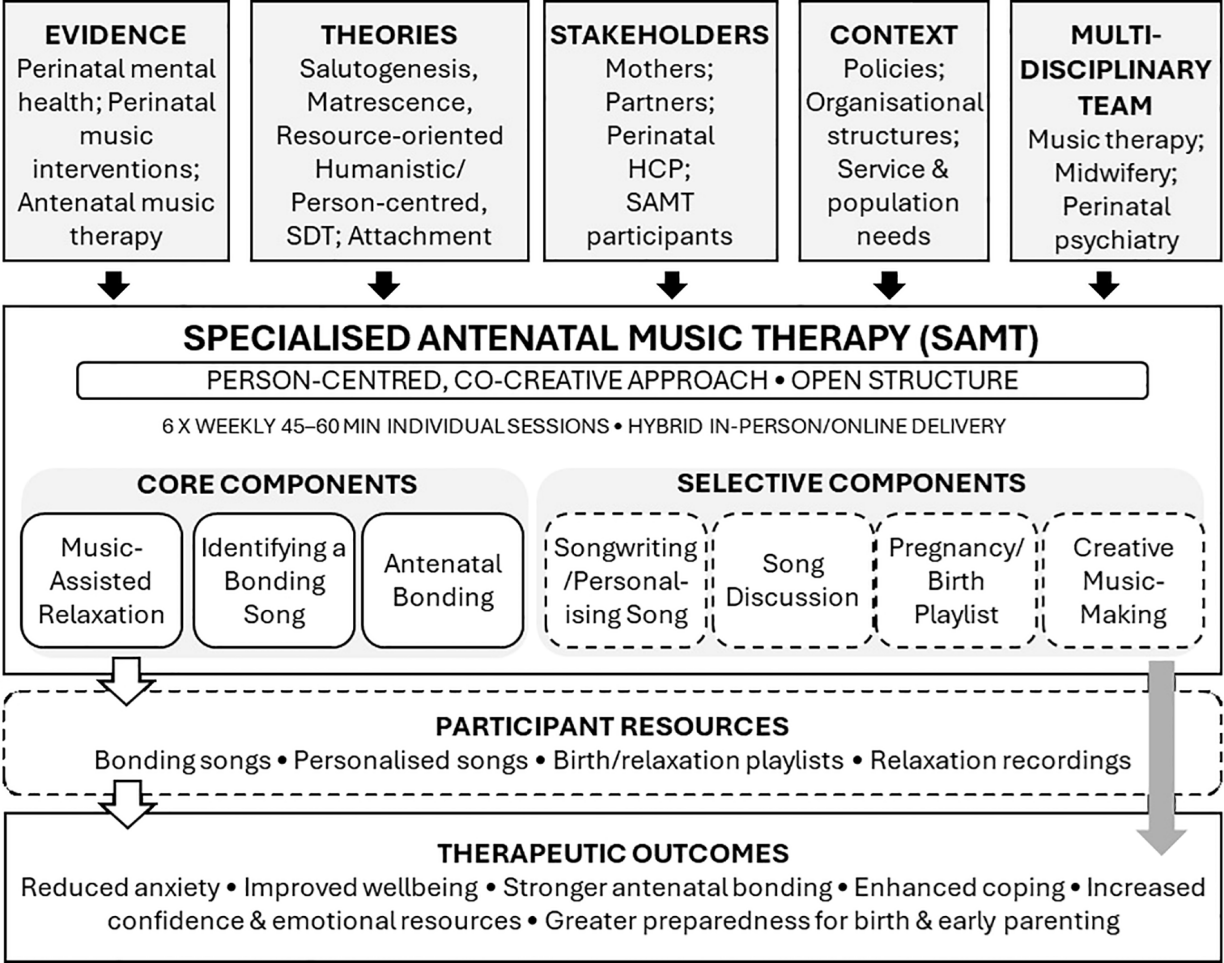
Logic model for the specialised antenatal music therapy (SAMT) intervention.

To consolidate and visually integrate the components described in this section, **Figure 1** presents a logic model of SAMT. The model illustrates how the intervention’s foundational inputs (evidence base, theoretical frameworks, stakeholder priorities, service context, and multidisciplinary expertise) inform its core design principles, including the person-centred, co-creative structure and flexible hybrid delivery. It also maps the core and optional therapeutic components, participant resources, and the intended short- and medium-term outcomes. This logic model provides a clear overview of the intervention to support transparency, replication, and future implementation within diverse perinatal mental health settings.

## 4 Discussion

This paper presents the development of the SAMT intervention, the first antenatal music therapy programme explicitly designed for women experiencing perinatal mental health difficulties and developed systematically using the MRC framework for complex interventions (31). By integrating the TIDieR checklist (29), the intervention is reported with sufficient clarity to support replication, fidelity assessment, and future adaptation. This contribution addresses a persistent gap in the literature, where most music therapy interventions lack detailed theoretical, procedural, and contextual description (93).

The development process combines empirical evidence, clinical expertise, and stakeholder engagement. This approach embodies key components of evidence-based practice models, which emphasise asking clinically relevant questions, acquiring and appraising evidence, applying findings to practice, and evaluating outcomes in collaboration with patients (94). A stakeholder-informed approach aligns with NICE guidelines that emphasise involving women, partners, and families in planning and decision-making throughout perinatal care, to improve acceptability, accessibility, and equity of interventions (95).

The developmental process also included feasibility and efficacy testing through a proof-of-concept study, which provided preliminary data that SAMT was acceptable, feasible, and therapeutically valuable. The successful referrals from the SPMHS team underscore the potential for other healthcare professionals such as GPs, obstetricians, midwives, and nurses to actively refer, support, and integrate SAMT within continuity-of-care frameworks. The findings are consistent with research demonstrating the benefits of music engagement for emotional regulation, bonding, and stress reduction during pregnancy (26, 39, 48, 96). These findings provide a robust foundation for the next stages of intervention refinement and evaluation. Priorities include a multisite feasibility trial, the development of a therapist training and supervision framework, assessment of intervention acceptability across diverse populations, and economic evaluation.

Interpreting the findings from the proof-of-concept study requires careful consideration of music therapy as a complex intervention rather than a standardised therapeutic “dose” of music. Complexity refers to the interdependence of multiple components, the importance of therapeutic relationships, the adaptive nature of delivery, and the influence of contextual factors on participant outcomes. DeNora and Ansdell (97) highlight that music’s influence is relational and situational, making variability in response an expected and clinically meaningful feature rather than a methodological problem. Instead, these varied responses offer valuable opportunities for therapists to engage in deeper therapeutic exploration through the therapeutic alliance that supports effective music therapy (98). From a therapeutic standpoint, these divergent responses are not barriers to fidelity but opportunities for exploration and meaning making within the therapeutic relationship. This perspective is consistent with established work on treatment manual development, which emphasises that complex relational interventions cannot function as rigid, standardised packages but must remain adaptable to individual clients, therapists, and contexts (99).

Recognising music therapy as a complex intervention also has methodological implications. This perspective challenges the assumption that individual musical activities can be treated as discrete, universally transferable “active ingredients.” As opposed to focusing on fixed techniques or activities (such as singing, songwriting, or improvisation), the open structure allows SAMT to be tailored for differing cultural contexts, delivery modes, and clinical populations without compromising fidelity. This echoes broader debates on balancing fidelity with contextual flexibility in music therapy and community music therapy (100). This adaptability positions SAMT as a scalable, woman-centred intervention suitable for implementation across varied maternity and mental health service settings. In this sense, SAMT contributes not only a novel antenatal music therapy protocol but also a model of how to conceptualise, implement, and evaluate music therapy interventions in a systemic way, aligning with the MRC framework (31).

## 5 Conclusion

Perinatal mental health is a global health concern that warrants urgent action to better support women, their families and society. SAMT provides a novel, theory-driven, and systematically developed model of antenatal music therapy tailored specifically for women experiencing psychological distress during pregnancy. By following the MRC framework and reporting the intervention comprehensively using the TIDieR checklist, this paper offers a transparent and replicable account of SAMT’s rationale, components, delivery, and theoretical foundations. SAMT contributes an important methodological advance for the field and establishes a clear foundation for future feasibility, effectiveness, and implementation research. As perinatal mental health services continue to expand, SAMT represents a promising, woman-centred therapeutic approach with potential for integration into specialist care and for adaptation across diverse clinical settings.

## Data Availability

The original contributions presented in the study are included in the article/supplementary material. Further inquiries can be directed to the corresponding author.
